# Normative data and comprehensive psychometric evaluation of the Hogg Eco-Anxiety Scale in a large Italian sample

**DOI:** 10.1016/j.heliyon.2024.e41406

**Published:** 2024-12-20

**Authors:** Giuseppina Spano, Elisabetta Ricciardi, Luigi Tinella, Alessandro O. Caffò, Giovanni Sanesi, Andrea Bosco

**Affiliations:** a Department of Psychology and Health Science, Pegaso Telematic University, Naples, Italy; b Department of Precision and Regenerative Medicine and Ionian Area, University of Bari, Bari, Italy; c Department of Humanities, Philosophy and Education, University of Salerno, Salerno, Italy; d Department of Educational Sciences, Psychology, Communication, University of Bari, Bari, Italy; e Department of Soil, Plant and Food Sciences, University of Bari, Bari, Italy

**Keywords:** Eco-anxiety, Climate anxiety, Cognitive reappraisal of climate crisis, Validation, Rasch analysis, Mental health

## Abstract

As the climate crisis intensifies, its impact on mental health becomes more apparent, leading to psychological distress such as eco-anxiety. Driven by growing scientific interest, standardized assessment tools for evaluating eco-anxiety are gaining traction. This study comprehensively validates the Hogg Eco-Anxiety Scale (HEAS) on a large sample of nearly 1300 participants across Italy and four generations. The scores obtained using the Italian HEAS demonstrate excellent reliability and support valid inferences regarding eco-anxiety levels. The original four-factor structure of the scale was upheld. Nonetheless, the scale proved to be reliable also in terms of a unique factor. Internal consistency was satisfactory, but stability over three months was not confirmed. The overall HEAS score was linked to pro-environmental behavior and reduced meat consumption. Additionally, the scale effectively identifies individuals engaged in public pro-environmental actions. Normative data were provided for age, gender, and education levels. These findings confirmed and extended the HEAS's validity and reliability, highlighting the importance of standardized assessment tools in this burgeoning research field and in clinical practice. Further exploration of its diagnostic efficacy in clinical settings for eco-anxiety detection is suggested.

## Highlights

• A complete validation of the Italian version of the HEAS is provided.
• HEAS-IT showed very good content, construct, and criterion validity and reliability.
• Eco-anxiety is linked to pro-environmental behavior and reduced meat consumption.
• Normative data across four generations, gender, and education levels are provided.
• 'Anxiety about the impact of personal behaviours' is a particularly sensitive item.

## 1 Introduction

Accumulated evidence is available on the direct and indirect effects of the climate crisis on the mental health of individuals and communities. The direct implications of the climate crisis impact populations at risk of ecological disasters or extreme effects of climate change such as floods, earthquakes, heatwaves, and fires [[1], [2], [3]]. Conversely, the indirect implications primarily stem from the awareness of the real and potential negative effects of extreme weather events on the environment and human health, which can affect even those who have no direct experience [4]. The psychological response to climate crisis, regardless of whether these are direct or indirect implications, is anxiety or other negative emotions such as worry, concern, grief, fear, and/or anger, with differential impact on general mental health and engagement in pro-environmental behavior [5,6]. Indeed, this also reflects a confusion in the terminology used to describe the phenomena. However, the range of emotions related to the climate crisis is more commonly referred to as "climate anxiety" or to "eco-anxiety". Although the most readily available studies have been conducted in Western countries [7], evidence is suggestive of a cross-national nature of such negative emotional responses to the climate crisis [8]. A cross-national study [9], conducted on a sample from 25 countries, highlighted that the negative emotions related to climate changes are moderately associated with subjective measures of mental health, including insomnia and self-rated general mental health. Another recent cross-national study [10] across 32 countries confirmed this finding, adding evidence of an association between climate anxiety and the tendency toward pro-environmental behavior and environmental activism, especially in wealthier countries.

From a generational perspective, the mental health of adolescents and young adults appears to be the most significantly impacted by the above-mentioned symptoms [7,11]. A recent scoping review (Ramadan et al., 2020) reported the presence of climate-related negative emotions also suggesting associations with mental health in young people. Evidence for a small gender effect was also found among young people aged 16–25 in 10 countries, reporting females showing higher levels of concern and negative emotions, whereas males were more optimistic and demonstrated greater faith in the government [12].

### 1.1 The assessment of climate anxiety or eco-anxiety

Nevertheless, is anxiety related to climate change a clinical condition or an expected psychological response to the climate crisis? To address this inquiry, it is useful to summarize the perspective of van Valkengoed [13], considering this phenomenon as a mental health issue rather than an expected response to the climate crisis. According to the author, normalizing this type of anxiety would not justify researchers' efforts in defining, measuring, and ultimately addressing it. As with any other mental health issue, the development and refinement of reliable diagnostic tools are imperative as a fundamental step to ensure early identification, effective treatment, enable scientific research, and enhance communication among mental health professionals. With the growing interest from researchers and clinicians, we are witnessing the proliferation of assessment tools, specifically scales. The *Climate Change Anxiety Scale* (CCAS – Clayton, & Karazsia, 2020) represents the first scale precisely developed for the assessment of climate anxiety and dates back not too long ago. CCAS dimensions are cognitive-emotional and functional impairment respectively assessing the capacity of the climate-related negative emotions to affect thinking (e.g., rumination) and emotions (e.g., mood) and to interfere with daily activities. An adapted short form of the CCAS for youth is also available [14]. Further available validated scales for constructs partially overlapping with climate change anxiety include, for example, the *Inventory of Climate Emotions* (ICE – [15]) which detect eight types of emotional responses to climate change, the *Climate Change Worry Scale* (CCWS - [16]), and the *Climate Change Distress Scale* [17] preceding others at a time when there was only a general discussion concerning stress and adverse emotional experiences in relation to environmental issues. In 2021, a rating scale for measuring eco-anxiety was developed and validated, the *Hogg Eco-Anxiety Scale* (HEAS – Hogg et al., 2021). The scale, consisting of 13 items distributed across four dimensions, was validated on a sample of individuals from Australia and New Zealand, demonstrating excellent reliability in its scores and supporting the validity of inferences made from these scores. It deviates from previous scales as it considers emotional and behavioral dimensions, introducing a distinct dimension never considered before, i.e., anxiety regarding personal impact on the planet. For this reason, the authors preferred using the term "eco-anxiety" as a broader concept than climate anxiety. International validations of the HEAS have been conducted, including adaptations in Portugal [18], Germany [19], and Turkey [20]. Preliminary evidence on the reliability of the scores and the validity of the inferences drawn from the HEAS was also investigated in an Italian sample of 335 adults [21]. The study provided descriptive data for gender (male vs. female) and for two age groups (≤30 vs. >30 years old). Similarly, Innocenti and colleagues [22] tested the factorial validity and internal consistency of the HEAS in a sample of 150 Italian adults. The two mentioned studies offer a significant contribution to the validation of the Italian adaptation of the HEAS and serve as a useful starting point for the present study. Despite the abovementioned studies, measures confirming the test's validity on larger samples are needed.

### 1.2 The present study

The overarching aim of this study is to complement previous preliminary validation work on the Italian version of the Hogg Eco-Anxiety Scale (HEAS-IT) by providing a comprehensive overview of the reliability of the scores and the validity of inferences drawn from the scale on a large sample of approximately 1300 participants located across the Italian peninsula and belonging to four generations, i.e., Baby Boomers (born from 1946 to 1964), Generation X (born from 1965 to 1980), Millennials (born from 1981 to 1996), and Generation Z (born from 1997 to 2012), classified according to the *Demographic outlook for the European Union* [23].

Results on content, factorial, construct, criterion validity, test-retest reliability, internal consistency, and Rasch analysis are provided. Furthermore, normative data for the Italian population across generation, gender, and education level are released for the first time, as a first step for exploring a diagnostic use of the scale.

## 2 Method

### 2.1 Total sample and procedure

This research project received ethical approval from the Human Research Ethics Committee of the local Department (Code No. ET-23-03).

Participants were recruited using a “snowball sampling” procedure. Study protocol was uploaded on an online platform (timeframe for data collection: from 9 January to February 8, 2023) and the link for its completion was spread among the so-called “initial participants”, who are part of the authors' social network. These initial participants are directly contacted and, upon completing the questionnaire, are invited to share the questionnaire link with other individuals within their social networks. A total of 1302 completed questionnaires were collected. Nine participants were excluded as they were under the age of 14, which we had set as the lower age limit. No upper age limit was set. Total sample resulted in 1293 participants, aged between 14 and 77 years (734 self-identified as females; 548 self-identified as males; 6 self-identified as non-binary people; 5 preferred not to answer). After briefly explaining the research objective and the regulations regarding anonymity and data processing through written text (see Supplementary Materials for details), each participant was asked whether they agreed to participate in the study by clicking 'I Agree'. If the participant did not click, the questionnaire was programmed not to proceed further, making its completion impossible. We obtained consent directly from adolescent participants (aged 14 years or older), a highly relevant sample for our study as the psychological effects of the climate crisis appear to be more keenly felt by this population target (Ramadan et al., 2020). Consistently with national guidelines and legislation in Italy concerning the protection of personal data, we selected 14 years as the cutoff age for participant inclusion. Specifically, Legislative Decree 101/2018, which aligns Italian law with the EU General Data Protection Regulation (GDPR), stipulates that the consent for processing personal data is valid from the age of 14; otherwise, consent from the holder of parental responsibility is required. This provision aligns with the guidelines of Data Protection Code's guidelines on processing personal data about children (https://www.linklaters.com/en/insights/data-protected/data-protected---italy) which outline the regulatory framework for processing minors' personal data in Italy.

### 2.2 Data analysis

Statistical analysis for study one, two, and three were analyzed using IBM SPSS Statistics (Version 24, IBM Corp, 2016) and JAMOVI 2.3 [24] for Windows.

## 3 Study one – content validity

Study 1 was aimed at analyzing content validity, which refers to the ability of the items scale in representing the theoretical construct (in this case, eco-anxiety). A set of 13 items indicated by Hogg et al. [25] in the original validation was tested using Lawshe's approach [26].

This method is widely recognized for content validation, yet it has several limitations, including potential subjectivity in expert judgment, lack of expertise weighting, and difficulties in generalizing findings to constructs with ambiguous boundaries. These limitations suggest that future studies may benefit from supplementing Lawshe's method with additional techniques, such as Item Response Theory (IRT) or Bayesian modeling, to capture a more nuanced evaluation of item content validity (AERA, APA, & NCME, 2014). However, we opted for interrater agreement as an initial validation step, given the scale's early stage in development.

A subset of the entire sample (N = 40) composed of master students was offered an in-depth training on the construct of eco-anxiety, including existing literature, common symptoms, causes, population at-risk, and potentially related clinical conditions. After the training, they were asked to judge each item scale as being “essential,” “useful but not essential,” or “not necessary”.

### 3.1 Measure

The **Hogg Eco-Anxiety Scale** (HEAS - [25]) was administered after translation of the items into Italian. Two native English speakers translated the original English items into Italian separately and resolved any discrepancies through discussion. The Italian items were later translated back into English by two other researchers who were not aware of the original scale. Lastly, the definitive wording for the Italian items was decided upon (Table S1 in Supplementary Materials). The scale is composed of 13 items with a Likert-type response scale ranging from (0) *not at all* to (4) *nearly every day.* The original scale demonstrated excellent score reliability and dimensional stability over time, supporting the validity of inferences drawn from the scale [25].

### 3.2 Results and discussion

Following the rule reported by Lawshe [26], indicating that items rated as “essential” by more than half of the judges has some degree of content validity, and thus must be included, no item was excluded by our set. Then, the Content Validity Ratio (CVR) index was applied, as follows: CVR=(NE-N/2)/(N/2) where NE is the number of judges indicating “essential” and N is the total number of judges. The final CVR computation is dependent by the number of panelists or experts who assess the items. The minimum CVR required with 40 judges is 0.29 (p = 0.05). CVR values of all the 13 items were higher than the cut-off, ranging from 0.45 to 0.85. The mean CVR across items was equal to 0.71 demonstrating a good content validity overall (Table 1).

**Table 1:** Content Validity Ratio (CVR) indicating overall test content validity (mean CVR) for the subsample of judges (N = 40).

*Items*	*CVR*
Q1. Feeling nervous, anxious or on the edge	0.70
Q2. Not being able to stop or control worrying	0.75
Q3. Worrying too much	0.45
Q4. Feeling afraid	0.75
Q5. Unable to stop thinking about future climate change and other global environmental problems	0.85
Q6. Unable to stop thinking about past events related to climate change	0.70
Q7. Unable to stop thinking about losses to the environment	0.55
Q8. Feeling anxious about the impact of your personal behaviours on the earth	0.85
Q9. Feeling anxious about your personal responsibility to hekp address environmental problems	0.75
Q10. Feeling anxious that your personal behaviours will do little to help fix the problem	0.80
Q11. Difficulty sleeping	0.65
Q12. Difficulty enjoying social situations with family and friends	0.70
Q13. Difficulty working and/or studying	0.75
*mean CVR*	**0.71**

Overall, our finding confirmed the content validity of the item translated into Italian originally composing the Hogg Eco-Anxiety Scale. In other words, the Italian version of the scale proved to measure the construct of “eco-anxiety”.

## 4 Study Two – dimensionality, construct and criterion validity

Considering the results of the Study 1, Study 2 was aimed at evaluating the construct and criterion validity of the 13-item scale. Exploratory Factor Analysis (EFA) and Confirmatory Factor Analysis (CFA) on independent samples were performed to identify and verify the factor structure of the scale, respectively, to determine whether the four-factor structure of the scale is replicated in the Italian version. Configural model tests for an overall similarity of factor structure across groups (i.e., generations, gender, and education) was performed using Multigroup Confirmatory Factor Analysis (MGCFA). Discriminant validity was evaluated by testing the association of self-reported eco-anxiety with self-reported spatial anxiety, while convergent validity through the association with climate change anxiety and worry. We expected eco-anxiety to be highly correlated with climate change anxiety and, to a lesser extent, with climate change worry, due to the similarity of the constructs related to a concern about the effects of climate change and environmental crisis. On the opposite, a low correlation is expected between eco-anxiety and spatial anxiety. Both constructs refer to a state of discomfort towards circumstances related to the external environment. However, in the first case, the reference is to ecological conditions (e.g., climatic issues) while, in the second case, to conditions relating to orientation in space (e.g., topographic disorientation). Heterotrait-Monotrait ratio of correlations (HTMT) was also calculated to assess the discriminant validity of latent variables of the scale.

Criterion – concurrent - validity was assessed in association of a set of three criteria: (1) participating in social media groups and/or pages (e.g., on Facebook, Instagram, Twitter) on news regarding environmental crisis and protection (i.e., “*digital activists*”), (2) actively participating in groups or associations for environmental protection (i.e., “*environmental activists*”), and (3) reducing or quitting meat consumption in the last year (i.e., “*meat reducers*”), [8]. A reduction of at least two types of meat including pork, poultry and beef was considered necessary to become part of the "meat reducers" group. We expected that self-reported eco-anxiety levels being able to discriminate at least the group of environmental activists and meat reducers and not the group of the digital activists as the implemented actions appeared to be not very demanding (e.g., following a social page and reading news by scrolling the feed). Also, the Average Variance Extracted (AVE) was calculated a measure of the amount of variance that is captured by a construct in relation to the amount of variance due to measurement error.

### 4.1 Method

#### 4.1.1 Participants

Three independent subsets were randomly selected by the total sample. Two for performing EFA and CFA analysis, respectively (EFA sample: N = 400; CFA sample: N = 500). The remaining one (N = 393) for performing convergent, discriminant, and criterion validity. Participants included in the EFA sample (296 females; 95 males; 6 non-binary people; 3 prefer not to answer) aged between 14 and 70 years (M_age_ = 30.62; SD_age_ = 13.76). Participants included in the CFA sample (250 females; 250 males) aged between 16 and 75 years (M_age_ = 35.84; SD_age_ = 16.38).

Sample size for both subsets of EFA and CFA in the present study was compatible with available best practices for exploratory and confirmatory factor analyses [27]; [28]. Specifically, for the confirmatory factor analysis (CFA), the sample size (N = 500) aligns with minimum guidelines for complex models to ensure adequate statistical power. Established recommendations, such as those by Satorra and Saris [29] and by Muthén and Muthén [30], support our selection, indicating that our sample size is sufficient for reliable parameter estimation within SEM frameworks.

Participant included in the third selected subset (188 females; 203 males; 2 prefer not to answer) aged between 18 and 77 years (M_age_ = 40.97; SD_age_ = 16.14).

#### 4.1.2 Measures

- *Eco-anxiety* was measured using the Italian version of the *Hogg Eco-Anxiety Scale* (HEAS - [25]) (see Study 1).
- *Spatial anxiety* was measured using the Italian translation of the *Spatial Anxiety Scale* (SAS - [31]). The scale assesses the presence of anxiety during eight everyday activities involving spatial and navigation abilities on a 5-point scale from “*not at all*” to “*very much*”, referring to the level of anxiety experienced. The higher the total score, the more anxiety the participant reported during wayfinding in unfamiliar environments. This scale was used to assess the discriminant validity of the HEAS.
- *Climate change anxiety* was measured using the Italian version of the *Climate Change Anxiety Scale* (CCAS - [32]) a self-report 13-item scale on a two-factor structure. Participants indicated the frequency in experiencing anxiety-related symptoms in situations connected with potential consequences of climate change on a 5-point Likert scale from (1) *never* to (5) *almost always.* The scale displayed good psychometric characteristics tested on a sample of 150 Italian adults.
- *Climate change worry* was measured using the Italian version of the *Climate Change Worry Scale* (CCWS –[33]), for assessing the level of concern related to climate change. The 10-item scale revealed a single-factor structure; response scale used was based on a 5-point Likert scale from(1) *never* to (5) *almost always.* The scale demonstrated good validity and reliability in assessing climate change worry in a sample of 13 Italian adults.

Alongside the scales, a set of items on demographics (e.g., age, gender, education) and information on activist behavior and meat consumption was also administered through the following multiple-choice questions: “Are you subscribed to any social media pages related to environmental news (e.g., on Instagram or Facebook?"; “Are you an activist for any environmental organization?"; “Did you reduce the amount of beef/pork/chicken you consumed over the past year?”.

#### 4.1.3 Results and discussion

**Fig. 1:**
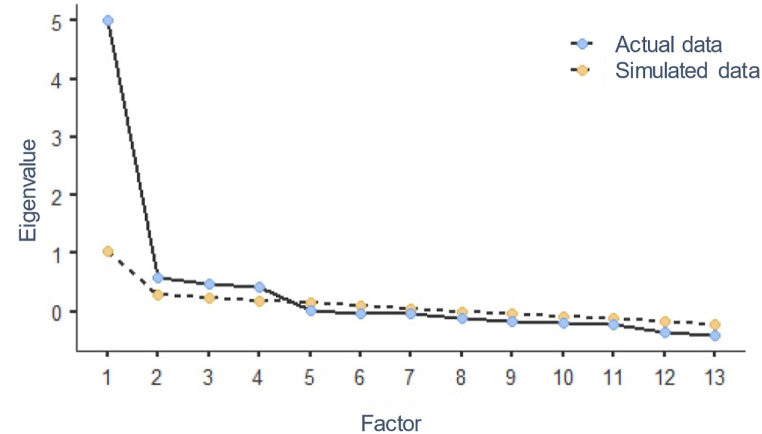
Scree plot from parallel analysis, illustrating the eigenvalues for each factor and the corresponding random data distribution.

After confirming that the assumptions of normality, linearity, homogeneity, and homoscedasticity were met, dimensionality of the 13-item eco-anxiety scale was explored on the EFA sample performing an Exploratory Factor Analysis (EFA) using *oblimin* rotation and *maximum likelihood* as extraction method. The number of factors was based on parallel analysis (Fig. 1). In line with the original validation, results revealed a clearly defined four-factor solution with a total variance explained of 57.3 % (Table 2). As expected, the first factor corresponds to feelings of anxiety and worry, explaining 16.9 % of the variance. The second factor (14.4 %) refers to repetitive contemplations regarding adverse environmental occurrences. The third factor (13.4 %) signifies concerns about one's individual impact on the Earth. The fourth factor (12.7 %) indicates observable indications of eco-anxiety in one's behaviour, such as struggles with sleep, work or study, and social interactions.

**Table 2:** Factor loadings from Exploratory Factor Analysis of the 13-item eco-anxiety scale from Study Two, EFA sample one (n = 400).

Item	Factor			
	Affective symptoms	Rumination	Anxiety about personal impact	Behavioral symptoms
Feeling nervous, anxious or on the edge	**0.714**	0.013	0.019	0.066
Not being able to stop or control worrying	**0.604**	0.127	0.004	−0.033
Worrying too much	**0.876**	−0.021	0.020	−0.038
Feeling afraid	**0.475**	0.035	0.005	0.210
Unable to stop thinking about future climate change and other global environmental problems	0.143	**0.578**	0.038	0.062
Unable to stop thinking about past events related to climate change	−0.059	**0.823**	0.031	−0.005
Unable to stop thinking about losses to the environment	0.034	**0.764**	−0.0339	−0.01310
Feeling anxious about the impact of your personal behaviours on the earth	0.145	−0.0101	**0.622**	−0.003
Feeling anxious about your personal responsibility to help address environmental problems	0.067	0.098	**0.481**	0.183
Feeling anxious that your personal behaviours will do little to help fix the problem	−0.0351	5.30e-4	**0.943**	−0.016
Difficulty sleeping	−0.046	0.020	0.04709	**0.802**
Difficulty enjoying social situations with family and friends	0.0378	−0.0122	−0.029	**0.859**
Difficulty working and/or studying	0.136	0.214	0.027	0.314

**Fig. 2:**
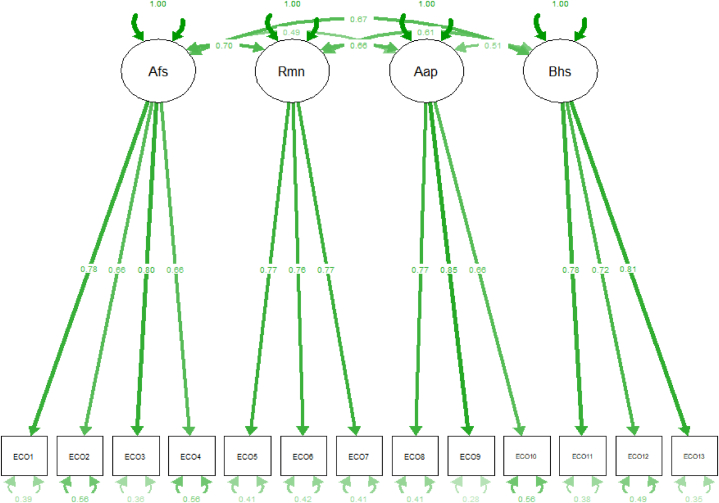
Four-Domain Confirmatory Factor Model of the Italian version of the Hogg Eco-Anxiety Scale: Standardized Estimates of Factor Loadings and Factor Covariances. *Note.* Afs = Affective symptoms: Rmn = Rumination; Aapi = Anxiety about personal impact; Bhs = Behavioral symptoms; It = Item.

To confirm the four-factor structure, a CFA was conducted with the other independent CFA sample. We tested a four-dimensional model as it emerged from the EFA and is the one that resulted in the original validation of the Hogg's scale. The model tested (Fig. 2) revealed excellent model fit indices: χ2(59) = 135 (p < 0.001), CFI = 0.973, TLI = 0.965, SRMR = 0.0362, RMSEA = 0.051 (90 % CI [0.04, 0.06], according to the guidelines provided by Hu and Bentler [34] and MacCallum and colleagues [35].

No modification techniques to the model were used for improving the fit indices. Our results confirmed the four-factor solution with the same distribution of items as identified by Hogg et al. [25], confirming the robust multidimensionality of the eco-anxiety construct, statistically established by the presence of four factors, namely affective symptoms, rumination, behavioural symptoms, and anxiety about one's personal impact on the planet also on an Italian sample.

Configural invariance across groups (Table S2) revealed that the configural invariance models had mixed fit to the data. The results indicate that the model is configurally invariant across gender groups, suggesting that the factor structure is similar for both. Configural invariance is approximately supported, indicating that the factor structure is similar across the three education groups. However, the slightly elevated RMSEA may suggest the need for model refinements or further verification at higher levels of invariance. Configural invariance is partially supported for generational groups, but the indices (particularly RMSEA) suggest that the model may not fit uniformly across all generations. Significant differences in sample sizes across groups (e.g., education level and generation) may introduce issues in invariance testing. Smaller groups can result in unstable parameter estimates, affecting the accuracy of model fit indices and potentially leading to biased results. For future studies, further invariance testing is recommended.

Discriminant and convergent validity of the scale were also evaluated. Both CCAS and CCWS were used for assessing the convergent validity of the HEAS-IT, whereas SAS for assessing discriminant validity. HEAS-IT and SAS scores exhibited a low and positive correlation (r = 0.23) while HEAS-IT and CCAS and CCWS scores exhibited satisfactory, positive correlations (r = 0.67 and r = 0.65, respectively). To confirm that the correlation between HEAS-IT and SAS was significantly different to the correlations between HEAS-IT and CCAS and HEAS-IT and CCWS, we performed a statistical comparison of the correlation triplets for dependent samples (HEAS-IT ∼ SAS: r = 0.23; HEAS-IT ∼ CCAS: r = 0.67; SAS ∼ CCAS: r = 0.20: test statistic Z = −8.481; p < 0.0001), (HEAS-IT ∼ SAS: r = 0.23; HEAS-IT ∼ CCWS: r = 0.65; SAS ∼ CCWS: r = 0.15: test statistic Z = −8.257; p < 0.0001). Our results are suggestive of a lack of overlapping between the constructs of eco- and spatial anxiety; while a moderate share of overlapping was found between eco-anxiety and climate change anxiety and worry, overall supporting the discriminant and convergent construct validity of the HEAS- IT scale.

Discriminant validity of the latent constructs of the scale was assessed using Heterotrait-Monotrait ratio of correlations (HTMT). All HTMT values are below the commonly accepted threshold of 0.85 (Affective symptoms and Anxiety about personal impact: HTMT = 0.612; Affective symptoms and Behavioural symptoms: HTMT = 0.663; Rumination and Anxiety about personal impact: HTMT = 0.706; Rumination and Behavioural symptoms: HTMT = 0.635; Anxiety about personal impact and Behavioural symptoms: HTMT = 0.530), indicating satisfactory discriminant validity among the constructs. This suggests that the scale is composed by well-differentiated factors, thus, that each construct appears to capture distinct aspects of the measured domain.In terms of criterion – concurrent - validity, as expected, results of logistic regressions showed that a stronger engagement in implementing pro-environmental actions, such as by giving charity to organizations for the defense of the biodiversity and participating in demonstrations to fight the climate crisis, was associated to higher level of eco-anxiety (β = 0.0825; *p* < 0.05). At the same time, the levels of eco-anxiety appeared to be discriminative between the group of those who had not reduced their meat consumption in the last year and those who had done so (β = 0.0570; *p* < 0.001), in line with our hypothesis. No association was found in relation to following social media groups (β = 0.0291; *p* = 0.089), thus supporting the hypothesis that eco-anxiety turns out to be a construct potentially associated to self-responsibility mechanisms and the implementation of pro-environmental behavior with a strong impact on daily life. These results are consistent with a recent cross-sectional study [8] conducted in China, India, Japan, and United States which found that climate change anxiety, measured using the Clayton's scale, was positively associated with climate activism and sustainable diet. This also contributes to further reinforcing the hypothesis that climate crisis-related anxiety is a global phenomenon.

In addition, the Average Variance Extracted (AVE) was calculated to verify convergent validity, with values exceeding the 0.50 threshold (ie., *Affective symptoms*: 53.3 %; *Rumination*: 58.9 %; *Behavioural symptoms*: 59.3 %; *Anxiety about personal impact*: 58 %), supporting that the constructs explain at least 50 % of the variance of its items. This is further reinforced by factor loadings greater than 0.60, indicating good convergent validity.

## 5 Study three –reliability and normative data

We tested internal consistency of the scale using Cronbach's Alpha (α), McDonald Omega (ω) coefficients and the Composite Reliability Index (CRI) for each factor. Above 0.7 is generally suggestive of an acceptable scale reliability value [36].

To assess the stability of eco-anxiety scores over time (n = 54), we calculated Intraclass Correlation Coefficient (ICC) estimates for the total scale and each subscale, based on a mean-rating (k = 2), absolute-agreement, 2-way mixed-effects model [37].

Furthermore, we performed a polytomous Rasch analysis [38]. Rasch analysis is a statistical method developed by Rasch [39] used to analyze the responses to a set of items to both measure an individual's ability, trait, or latent construct, and evaluate the quality of the items or questions used in the assessment. In our analysis, we examined both infit and outfit statistics for items. Item fit statistics indicate how well each item aligns with the overall scale. The standardized infit statistic is particularly sensitive to responses that deviate from the expected pattern near an individual's ability level, such as an incorrect score that falls below their actual ability. On the other hand, the outfit statistic is more sensitive to unexpected responses that are farther from the individual's measurement level, like an incorrect response to the easiest task or a correct response to a more challenging task [40]. Both infit and outfit statistics are expected to have an average value of 0 and a standard deviation of 1. When these fit statistics exceed 2, it signals a lack of alignment between the item and the proposed scale, a condition known as misfit; conversely, if the fit value is < −2, it suggests overfitting, indicating that the scale is overly deterministic compared to what the Rasch model predicts. Infit and outfit values close to 1 are indicative of a good fit between the theoretical model and the observed data [40]. Additionally, a A Wright map and a Person-Item map were produced. They provide a graphical representation of the distribution of item difficulties and person abilities on the same measurement scale map in order to assess how well the items in an assessment are aligned with the abilities of the individuals taking the test.

Lastly, the mean score, standard deviation, median score, InterQuartile Range (IQR), 90th and 95th percentiles, minimum and maximum scores, skewness, and kurtosis values were calculated for 19 out of 24 total categories by combining age, gender, and education starting from the total available sample.

### 5.1 Participants and procedure

Internal consistency and Rasch model were performed on the total sample (see Study One for demographics). Stability over time was calculated through a test-retest procedure on a self-selected subsample of the total one. Three months after the data collection, a mail was sent to each participant in order to allow a second online administration of the scale. Availability to be recontacted were collected during the first stage of administration and no participant had expressed refusal to be involved in the procedure. Fifty-four questionnaires were completed during the retest phase.

### 5.2 Results and discussion

Reliability was calculated on the total sample. The scale showed excellent internal reliability (α > 0.891; ω > 0.894) if considered as a unidimensional scale. Item reliability statistics also confirm that all items in the scale deserve inclusion. In other words, no item, if discarded, contributes to the increase in the internal consistency coefficients. Composite Reliability Index (CRI) values indicate a good level of reliability for each factor (ie. Affective symptoms: 0.819; Rumination: 0.811; Behavioural symptoms: 0.814; Anxiety about personal impact: 0.804), as a CRI above 0.7 is generally considered acceptable for psychometric instruments. All indices were suggestive of a robust internal consistency for the scale.

In contrast with Hogg and colleagues [25], we found weak ICCs for all the subscales (affective symptoms: ICC = 0.102, 95 % CI [−0.06,0.336]; behavioural symptoms: ICC = 0.107, 95 % CI [−0.05,0.352]; rumination: ICC = 0.158, 95 % CI [−0.07,0.446]; personal impact anxiety: ICC = 0.125, 95 % CI [−0.08,0.359]) and for the total scale (ICC = 0.105, 95 % CI [−0.05,0.351]).

Therefore, no particular differences were found in the stability over time among the HEAS-IT dimensions, while a certain variability were found in the original validation where dimensions related to rumination and personal impact anxiety were found to be more stable than behavioural and affective symptoms [25].

In summary, contrary to what stated in the original validation, we found that none of the eco-anxiety dimension scores is stable over a 12- week period. The HEAS instructions refer to a series of sensations possibly experienced during the previous two weeks. This makes the scale looks more like a “state anxiety scale” indicating a measure an individual's temporary or situational level of anxiety instead of a stable personality characteristic [41]. Nonetheless, further exploration of test-retest reliability of the scale is warranted to disentangle this aspect, for example, enlarging the sample size, considering that in the original validation, a total of 189 participants responded to the follow-up.

The fit statistics of the items (Table 3) were found to be satisfactory. The values of infit and outfit are close to 1, indicating good fit between the theoretical model and the observed data.

**Table 3:** Fit statistics of the items.

	Measure	Standard Error Measure	Infit	Outfit
Q1. Feeling nervous, anxious or on the edge	1.010	0.0442	0.788	0.794
Q2. Not being able to stop or control worrying	1.918	0.0500	1.245	1.239
Q3. Worrying too much	1.672	0.0482	0.981	0.951
Q4. Feeling afraid	1.484	0.0470	0.958	0.938
Q5. Unable to stop thinking about future climate change and other global environmental problems	1.667	0.0482	1.016	0.944
Q6. Unable to stop thinking about past events related to climate change	2.207	0.0522	0.951	0.885
Q7. Unable to stop thinking about losses to the environment	1.462	0.0468	0.956	0.973
Q8. Feeling anxious about the impact of your personal behaviours on the earth	0.896	0.0437	1.025	1.007
Q9. Feeling anxious about your personal responsibility to help address environmental problems	0.934	0.0438	1.063	1.056
Q10. Feeling anxious that your personal behaviours will do little to help fix the problem	1.041	0.0444	1.198	1.169
Q11. Difficulty sleeping	3.859	0.0710	1.286	1.063
Q12. Difficulty enjoying social situations with family and friends	3.052	0.0601	0.979	0.951
Q13. Difficulty working and/or studying	3.483	0.0654	1.167	1.059

**Fig. 3:**
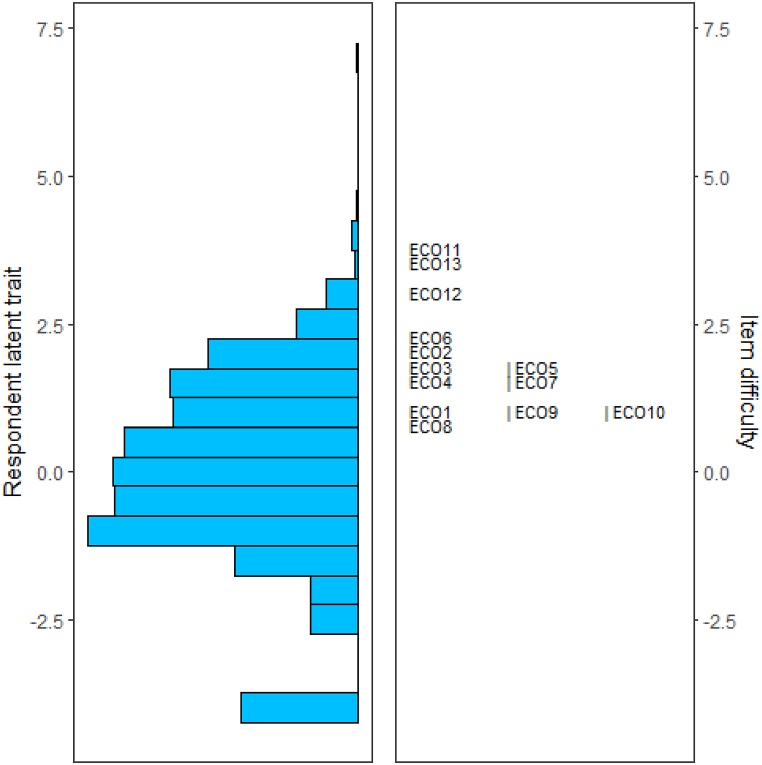
Wright item-person map indicating respondent ability and item difficulty in a Rasch analysis.

Fig. 3 shows the person and item threshold locations graphically. Given that in polytomous Rasch analysis, both individual and item parameters are estimated on a uniform scale, the evaluation of the alignment between individuals and items becomes feasible. A clear alignment suggests a good fit between item and respondent characteristics, contributing to the reliability of the measurement. As shown, the items polarize towards the upper end of the distribution of item difficulty, while participants are normally distributed but with a low average level of ability, that we must consider in terms of the tendency to endorse the questionnaire items. This confirms the hypothesis that the Hogg's scale can indeed be regarded as a symptom scale, and therefore, it might be used efficiently in a clinical context. In fact, in our sample based on the general, non-clinical population, participants exhibit low scores on the total scale (indicating the absence or low level of symptoms).

The Person-Item Map (Fig. S1) shows the distribution of respondents' abilities (left side, in gray bars) alongside item difficulty thresholds (right side, red markers) across a shared latent dimension. The spread of person abilities is generally broader than the item difficulties, suggesting some items may not fully cover the ability range. Our analysis of item spacing revealed areas with potential item overlap, suggesting similar content for some items (e.g., ECO1 and ECO10). We also identified significant gaps in item distribution (e.g., ECO6 to ECO12). To improve measurement precision, additional items spanning a wider difficulty range could be introduced to better match the full spectrum of respondent abilities. Revising items with narrow thresholds may also enhance discrimination by adding response categories that cover broader levels of difficulty.Analyzing the standardized mean scores of groups divided according to criterion 2, namely “environmental activism” (see Study Two for details), we identified an item with a high discriminative power for detecting 'environmental activists' (−0.037) and 'non-activists' (0.920). The identified item is the following: “*Feeling anxious about the impact of your personal behaviours on the earth*”. This item resulted to be highly endorsable for the group of environmental activists and low endorsable for non-activists. This finding is particularly interesting in light of the fact that the item fits in the dimension relating to anxiety about one's negative impact on the planet. This dimension is a newly introduced dimension that characterizes and distinguishes the Hogg's scale from previous climate anxiety scales, which instead only took into consideration the emotional and cognitive dimensions. We can hypothesize that a concern due to personal negative impact on the planet may promote pro-environmental behaviour, such as waste reduction, energy savings, and sustainable transportation [42].

Table 4 presents normative data on the overall sample, divided into a total of 19 participant subgroups, stratifying by generation, gender (male/female), and educational level. For sample sizes (see column “N” in Table 4) between 15 and 29 units, it is advised to handle the data with caution. Normative data for five subgroups of participants, i.e., males belonging to the “Baby Boomer” generation with less than 8 years of education, males and females belonging to “Millennials” or to “Gen Z” with less than 8 years of education, are not available due to a too little sample size.

**Table 4:** Normative data for eco-anxiety total score distributed across age (four generations), gender (two categories), and schooling (three levels).

Generation	Gender	Schooling	N	Mean Score	SD	Median	InterQuartile Range (IQR)	90th percentile	95th percentile	Sum Score	Minimum Score	Maximum Score	Skewness	Skewness Standard Error	Kurtosis	Kurtosis Standard Error
Baby Boomer	Female	≤8 years	16	24.3	9.84	23.5	13.5	38.0	41.0	388	13	44	0.608	0.564	−0.552	1.09
		9–13 years	20	24.6	7.04	24	10	34.1	35.0	491	13	36	0.0793	0.512	−0.951	0.992
		>13 years	17	24.8	8.60	23	8	35.2	39.2	421	14	48	1.34	0.550	2.18	1.06
	Male	≤8 years	12	–	–	–	–	–	–	–	–	–	–	–	–	–
		9–13 years	16	18.6	5.69	16.5	11	25.0	26.5	297	13	31	0.764	0.564	−0.463	1.09
		>13 years	22	21.8	7.85	20	11	31.9	35.8	480	13	40	0.818	0.491	−0.108	0.953
Generation X	Female	≤8 years	38	24.2	6.97	23.5	11	35.3	36.3	921	13	39	0.382	0.383	−0.502	0.750
		9–13 years	120	24.1	7.02	23	8.5	33.1	37.0	2895	13	48	0.815	0.221	0.826	0.438
		>13 years	104	23.1	6.46	22	9	30.0	36.4	2401	13	44	0.896	0.237	0.947	0.469
	Male	≤8 years	20	21.1	7.75	19	7	32.3	35.4	422	13	42	1.38	0.512	1.67	0.992
		9–13 years	67	20.4	5.24	19	7	28.0	29.7	1368	13	36	0.974	0.293	0.603	0.578
		>13 years	54	20.9	5.82	21	6.75	27.7	31.3	1128	13	37	0.719	0.325	0.664	0.639
Millennials	Female	≤8 years	3	–	–	–	–	–	–	–	–	–	–	–	–	–
		9–13 years	46	23.2	6.92	22	10.5	33.0	35.8	1067	13	39	0.520	0.350	−0.544	0.688
		>13 years	67	22.0	6.04	22	9	29.4	31.0	1471	13	40	0.817	0.293	0.695	0.578
	Male	≤8 years	2	–	–	–	–	–	–	–	–	–	–	–	–	–
		9–13 years	34	20.8	5.51	19.5	7	27.4	31.3	707	13	34	0.612	0.403	−0.137	0.788
		>13 years	27	21.1	6.60	19	8	30.6	33.0	571	13	38	0.852	0.448	0.391	0.872
Generation Z	Female	≤8 years	2	–	–	–	–	–	–	–	–	–	–	–	–	–
		9–13 years	146	23.4	5.44	23	8	31.0	33.0	3423	13	45	0.623	0.201	0.626	0.399
		>13 years	155	22.8	5.48	23	7	30.6	32.3	3539	13	39	0.630	0.195	0.404	0.387
	Male	≤8 years	10	–	–	–	–	–	–	–	–	–	–	–	–	–
		9–13 years	172	21.6	6.19	20	8.25	29.0	32.0	3720	13	52	1.26	0.185	2.88	0.368
		>13 years	112	21.3	6.05	20	9	28.0	33.0	2384	13	42	0.874	0.228	0.597	0.453

*Note*. Normative data for the gender categories 'self-identified as non-binary people' and 'preferred not to answer' were not calculated due to a low sample size. Empty rows indicate that the data was deemed unreliable due to a low sample size (<15). For sample sizes between 15 and 29 units, it is advised to handle the data with caution. For categories exhibiting a skewness below 1, the use of percentiles is recommended.

## 6 General discussion

The present study aimed to provide a comprehensive overview of the reliability of the scores and the validity of inferences drawn from the Italian version of the HEAS-IT in a large sample of Italians located in each region and covering four generations, i.e., Baby Boomer, Generation X, Millennials, and Generation Z. Previous research [21] showed that the Italian version of the HEAS-IT showed the same factor structure of the original validation, as well as a good internal consistency and a satisfactory convergent validity of the HEAS-IT with the validated Italian version of the Climate Change Anxiety Scale (CCAS – Clayton & Karazsia, 2020). Our results are perfectly in line with the Italian study and with the original validation [25], confirming the multidimensionality of the scale in four underlying factors, i.e., affective symptoms, rumination, cognitive symptoms, and anxiety about one's negative impact on the planet. Furthermore, our results confirmed previous evidence on concurrent validity and internal consistency. In addition to these findings, our study complements the psychometric analysis of the Italian version of the HEAS suggesting the discriminant construct validity with spatial anxiety, test-retest reliability and applying item-response-theory using polytomous Rasch analysis. We verified that the Italian version of the HEAS- IT showed excellent psychometric properties, including strong reliability of the scores and support for the validity of inferences drawn from the scale.

We endeavored to progress further. In the original study, it emerges how challenging it is to differentiate clinical and sub-clinical forms of eco-anxiety. However, it becomes complicated to statistically determine that the scale is capable of discriminating between clinical and non-clinical forms, as there is currently a lack of criteria defining a genuinely 'ill' sample due to eco-anxiety. From our perspective, we wondered whether by administering the scale to a non-clinical general population (confirmed by the fact that the HEAS score distribution of our sample remained rather low in terms of 'presence of symptoms'), it might be possible to intercept instead positive, adaptive, and pro-environmental behavior through the scale. On this regard, we found that the scale is able to detect individuals who are engaged in public pro-environmental behavior.

Engaging in behaviors that promote the well-being of the environment is crucial for tackling issues like climate change, pollution, and the decline of biodiversity [43]. When people embrace and advocate for these actions, they become pivotal contributors to the establishment of a world that is more sustainable and environmentally conscious. Furthermore, when communities collectively undertake pro-environmental initiatives, they actively contribute to larger endeavors aimed at attaining global environmental objectives and fostering a sustainable future for our planet [43]. Previous research shown that anxiety related to climate change has a dual impact on shaping Pro-Environmental Behaviors (PEBs). It can either prompt individuals to directly address their emotions and channel their efforts into PEBs [9]; [5,44,45], or it can lead to eco paralysis and a complete lack of initiative in response to climate change [22].

Switching the perspective, other studies reported that individuals who take environmental issues seriously and have firsthand experience with climate change are more likely to experience climate anxiety [11,46,47]. It's worth investigating whether people with PEBs are at-risk for developing symptoms of anxiety and/other eco-emotions related to consequences of climate crisis, such as eco-anger [48] and eco-worry [49]. For these, and other potentially at-risk groups, it is necessary to use a scale for diagnostic purposes in order to cope with this mental health problems. In this regard, indeed, although some at-risk populations, such as adolescents or young adults, seem to be more informed and aware of the disastrous effects of the climate crisis, they also appear to have lower levels of self-efficacy and higher levels of hopelessness [50,51].

To the best of our knowledge, this is the first study providing normative values for the Italian version of the HEAS, thereby constraining the utility of the scale as a screening tool in both research and clinical settings.

This work is not without limitations. For certain categories of individuals within the general sample, normative data are not available. This is especially true for individuals with low educational attainment (<8 years). It is important to note that for the two most recent generations, namely Millennials and Gen X, the minimum educational levels reach at least middle school education, making it challenging to obtain a sufficient sample size to provide normative data for psychological scales. Future research should certainly consider this aspect, particularly for validations conducted in industrially developing countries. Similarly, normative data is not provided for genders other than the binary. Although this information has been collected, we were unable to achieve a sufficient sample size in this case as well. Another limitation is the lack of a clinical sample. We recommend further studies that examine clinical samples (e.g., individuals diagnosed with generalized anxiety disorder) to test the diagnostic effectiveness of the scale in detecting potentially related symptoms and/or psychopathological disorders.

In addition, if participants were recruited through a self-selection method, there may be biases related to the fact that only certain individuals (e.g., those more motivated or more interested in the research) chose to participate. This could limit the generalizability of the findings to the broader population. Efforts to engage less motivated individuals or those with lower interest in the research topic may help improve generalizability.

From a statistical point of view, we employed interrater reliability to examine agreement among judges. However, in line with current standards in educational and psychological testing, content validity should ideally encompass broader evidence, including alignment of item content with the construct being measured, as well as item wording, themes, and administration procedures (AERA et al., 2014, pp. 11–31). This study aimed to evaluate initial content validity, thus we opted for using Lawshe's method and interrater reliability which offer valuable insights, nonetheless, further validation steps, such as IRT analyses, are recommended. In general, the findings of this study have significant implications for both environmental psychology and mental health. By confirming the psychometric robustness of the HEAS-IT, our work highlights its potential as a tool for identifying individuals experiencing eco-anxiety, a growing psychological concern linked to climate change. Moreover, the provision of normative data for the Italian population adds valuable reference points for future research and clinical applications, making the scale more accessible and useful for identifying individuals at risk of eco-related mental health issues. In fact, this scale can be applied not only in research settings to study the emotional impacts of environmental stressors but also in clinical contexts to detect at-risk individuals and guide interventions. Mental health professionals can use the HEAS-IT to assess eco-anxiety, which could contribute to the development of tailored therapeutic strategies. Moreover, understanding how emotional responses to climate change influence pro-environmental behaviours enables us to design interventions that both reduce psychological distress and promote sustainable actions. As eco-anxiety becomes an increasingly recognized mental health issue, the HEAS-IT has the potential to bridge the gap between environmental psychology and mental health, fostering greater interdisciplinary collaboration to address the psychological toll of climate change.

## 7 Conclusions

The intricate connection between the climate crisis and mental health is increasingly evident, with environmental challenges giving rise to diverse psychological reactions. A surge in anxiety, depression, and eco-anxiety reflects the uncertainties and repercussions of climate change. Acknowledging and tackling the intersection of climate issues and mental health is vital for devising comprehensive strategies to alleviate the human impact of the ongoing environmental crisis. Notably, our findings extend beyond prior studies by establishing the validity and reliability of the scale as a potential screening tool.

Improving or refining the reliability of the scores and the validity of the inferences drawn from a diagnostic scale can have significant positive implications for both scientific research and clinical practice. Enhancements in reliability and validity contribute to more consistent and accurate measurements, fostering increased confidence in research findings, applicability of the scale, and diagnostic decisions. These improvements also enhance the sensitivity, specificity, and, ultimately leading to standardized and widely accepted tools in the scientific community.

While challenges persist in distinguishing clinical and sub-clinical forms of eco-anxiety, our exploration into the positive, adaptive aspects of the scale revealed its ability to identify individuals engaged in pro-environmental behavior. We recommend further investigations involving clinical samples to assess the diagnostic efficacy of the scale in detecting symptoms and psychopathological disorders, thereby enhancing its utility in both research and clinical contexts.

## CRediT authorship contribution statement

**Giuseppina Spano:** Writing – review & editing, Writing – original draft, Methodology, Investigation, Formal analysis, Data curation, Conceptualization. **Elisabetta Ricciardi:** Writing – review & editing, Writing – original draft, Investigation, Data curation. **Luigi Tinella:** Writing – review & editing, Investigation. **Alessandro O. Caffò:** Writing – review & editing. **Giovanni Sanesi:** Writing – review & editing, Supervision. **Andrea Bosco:** Writing – review & editing, Supervision, Methodology, Funding acquisition, Conceptualization.

## Ethics approval and consent to participate

The present research received approval from the ethics committee of the Human Research Ethics Committee of the University of Bari with code ET-23-03 before its implementation. Throughout the research, ethical considerations such as confidentiality, honesty, and the participants' right to consent were upheld. At the outset of the study, the research objectives were communicated to the participants, and they were asked to agreed to participate in the study by clicking 'I Agree'. If the participant did not click, the questionnaire was programmed not to proceed further, making its completion impossible. We obtained consent directly from adolescent participants (aged 14 years or older), a highly relevant sample for our study as the psychological effects of the climate crisis appear to be more keenly felt by this population target (Ramadan et al., 2020). Consistently with national guidelines and legislation in Italy concerning the protection of personal data, we selected 14 years as the cutoff age for participant inclusion. Specifically, Legislative Decree 101/2018, which aligns Italian law with the EU General Data Protection Regulation (GDPR), stipulates that the consent for processing personal data is valid from the age of 14; otherwise, consent from the holder of parental responsibility is required. This provision aligns with the guidelines of Data Protection Code's guidelines on processing personal data about children (https://www.linklaters.com/en/insights/data-protected/data-protected---italy) which outline the regulatory framework for processing minors' personal data in Italy.

## Availability of data and materials

The datasets analyzed during the current study are available from the corresponding author upon reasonable request.

## Funding

G.Sp. (first author) was supported by the project “Role of PSychological and social factors in the conservation of biodivErsity and the ECOsystemic heritage for the fight against climate change (PsEEco)”, action co-funded by Programma Operativo (PON) Ricerca E Innovazione 2014–2020 – Azione IV.6 “Contratti di ricerca su tematiche Green” (CUP H95F21001420006). E.R. was supported by the project “EN.G.AGE. – The linking between ENvironment and coGnitive AGEing”. Action Co-founded by POR Puglia FESR FSE 2014–2020 - Asse X - Azione 10.4. (CUP H96D20000320008). AB was supported by the project “Testing the efficacy of remote, sustainable empowerment protocols in promoting psycho-physical well-being in the life-span” action funded by “Fondo per il Programma Nazionale di Ricerca e Progetti di Rilevante Interesse Nazionale (PRIN) 2022” (CUP 202284WCP9).

## Declaration of competing interest

The authors declare that they have no known competing financial interests or personal relationships that could have appeared to influence the work reported in this paper.
