# Supplementary material for: Normative data and comprehensive psychometric evaluation of the Hogg Eco-Anxiety Scale in a large Italian sample
Source: Heliyon. 2024 Dec 20;11(1):e41406. doi: 10.1016/j.heliyon.2024.e41406 (PMC11825253; doi:10.1016/j.heliyon.2024.e41406)
Supplement: Multimedia component 1 [file mmc1.docx]

**Supplementary material to the paper**

**“Normative data and comprehensive psychometric evaluation of the Hogg Eco-Anxiety Scale in a large Italian sample”**

**Table S1. Italian version of the Hogg Eco-Anxiety Scale (HEAS-IT)**

Istruzioni: “*Negli ultimi tempi, i temi legati al cambiamento climatico e ad altre condizioni ambientali globali (ad esempio, riscaldamento globale, degrado ecologico, esaurimento delle risorse, estinzione delle specie, buco dell’ozono, inquinamento degli oceani, deforestazione) sono al centro dell'attenzione.* ***Pensando a questi argomenti, NELLE ULTIME DUE SETTIMANE, quanto spesso si è sentito/a...****”*

| 1. | nervoso/a, ansioso/a, agitato/a? |
| --- | --- |
| 2. | non in grado di fermare o controllare le preoccupazioni? |
| 3. | troppo preoccupato/a? |
| 4. | spaventato/a? |
| 5. | come se fosse impossibile smettere di pensare ai futuri cambiamenti climatici e ad altri problemi ambientali globali? |
| 6. | incapace di smettere di pensare agli eventi passati legati ai cambiamenti climatici? |
| 7. | incapace di pensare alle perdite subite dall’ambiente? |
| 8. | in ansia per l’impatto dei miei comportamenti sulla Terra? |
| 9. | in ansia riguardo alla mia responsabilità personale nel contribuire ad affrontare i problemi ambientali? |
| 10. | per il fatto di pensare che i miei comportamenti personali non contribuiranno a risolvere il problema? |
| 11. | Pensando a questi temi, quante volte ti è capitato di avere difficoltà a dormire? |
| 12. | Pensando a questi temi, quante volte ti è capitato di avere difficoltà a godere delle situazioni sociali con la famiglia e gli amici? |
| 13. | Pensando a questi temi, quante volte ti è capitato di avere difficoltà a lavorare e/o studiare? |

Scala di risposta: 0 = per niente, 1 = per più giorni, 2 = più della metà dei giorni, 3 = quasi ogni giorno.

**English (back-translated) version of the Hogg Eco-Anxiety Scale (HEAS)**

**Instructions:** "In recent times, topics related to climate change and other global environmental conditions (e.g., global warming, ecological degradation, resource depletion, species extinction, ozone depletion, ocean pollution, deforestation) have been in the spotlight. **Thinking about these issues, IN THE PAST TWO WEEKS, how often have you felt...**"

| 1. | Nervous, anxious, agitated? |
| --- | --- |
| 2. | Unable to stop or control your worries? |
| 3. | Overly worried? |
| 4. | Frightened? |
| 5. | As if it were impossible to stop thinking about future climate changes and other global environmental issues? |
| 6. | Unable to stop thinking about past events related to climate change? |
| 7. | Unable to stop thinking about the losses suffered by the environment? |
| 8. | Anxious about the impact of your actions on the Earth? |
| 9. | Anxious about your personal responsibility in helping to address environmental issues? |
| 10. | From thinking that your personal actions will not help solve the problem? |
| 11. | **Thinking about these issues, how often have you experienced difficulty sleeping?** |
| 12. | **Thinking about these issues, how often have you experienced difficulty enjoying social situations with family and friends?** |
| 13. | **Thinking about these issues, how often have you experienced difficulty working and/or studying?** |

Likert scale: 0 = Not al all, 1 = Several days, 2 = More than half of the days, 3 = Nearly every day.

**Table S2. Main fit indices (CFI, TLI, RMSEA, SRMR) resulting from the configural invariance of factor structure) for the different groups (gender, education level, generation).**

|  | **Chi-square (df)** | **CFI** | **TLI** | **RMSEA (90% CI)** | **SRMR** | **Sample Size** |
| --- | --- | --- | --- | --- | --- | --- |
|  |  |  |  |  |  |  |
| **Gender** |  |  |  |  |  |  |
| Male | 110.009 (118) | 0.970 | 0.961 | 0.053 (0.040–0.066) | 0.045 | 250 |
| Female | 91.514 (118) |  |  |  |  | 250 |
|  |  |  |  |  |  |  |
| **Education** |  |  |  |  |  |  |
| Low | 103.051 (177) | 0.949 | 0.933 | 0.072 (0.060–0.084) | 0.049 | 52 |
| Medium | 102.472 (177) |  |  |  |  | 247 |
| High | 123.215 (177) |  |  |  |  | 201 |
|  |  |  |  |  |  |  |
| **Generation** |  |  |  |  |  |  |
| Baby Boomers | 115.066 (236) | 0.931 | 0.909 | 0.084 (0.072–0.096) | 0.060 | 33 |
| Generation X | 111.890 (236) |  |  |  |  | 173 |
| Millennials | 71.960 (236) |  |  |  |  | 45 |
| Generation Z | 145.220 (236) |  |  |  |  | 249 |
|  |  |  |  |  |  |  |


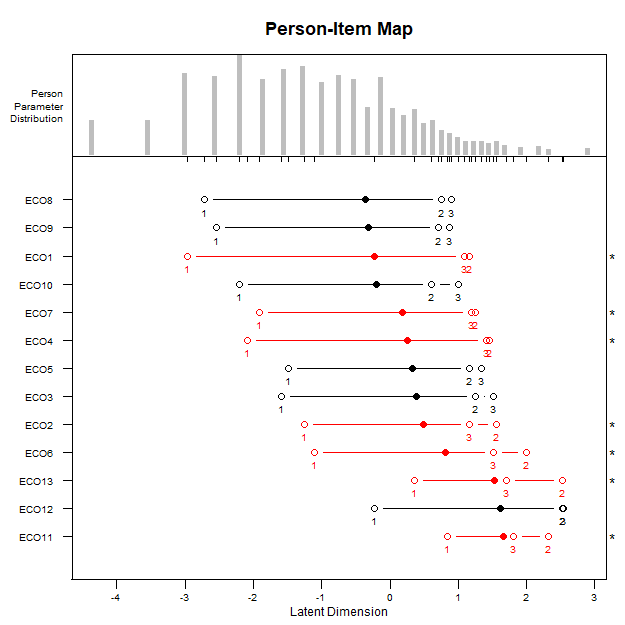


**Figure S1.** **Person-item map for Partial Credit Model (PCM) analysis.**
